# Autologous Stem Cell Transplant for AL Amyloidosis

**DOI:** 10.1155/2012/238961

**Published:** 2012-05-16

**Authors:** Vivek Roy

**Affiliations:** Division of Hematology/Oncology, Mayo Clinic, 4500 San Pablo Road, Jacksonville, FL 32224, USA

## Abstract

AL amyloidosis is caused by clonal plasma cells that produce immunoglobulin light chains which misfold and get deposited as amyloid fibrils. Therapy directed against the plasma cell clone leads to clinical benefit. Melphalan and corticosteroids have been the mainstay of treatment for a number of years and the recent availability of other effective agents (IMiDs and proteasome inhibitors) has increased treatment options. Autologous stem cell transplant (ASCT) has been used in the treatment of AL amyloidosis for many years. It is associated with high rates of hematologic response and improvement in organ function. However, transplant carries considerable risks. Careful patient selection is important to minimize transplant related morbidity and mortality and ensure optimal patient outcomes. As newer more affective therapies become available the role and timing of ASCT in the overall treatment strategy of AL amyloidosis will need to be continually reassessed.

## 1. Introduction

Amyloidosis is a disease of protein misfolding in which the involved protein acquires an abnormal beta-pleated sheet configuration rather than the native alpha helical state [1]. The amyloid protein is insoluble, and its deposition in various tissues causes tissue damage and organ dysfunction. Amyloidosis can be of various types based on the precursor protein involved in amyloid formation. More than 20 different human fibrillar amyloid proteins have been described [2], and accurate identification of the type is crucial to treatment planning. It is also important to distinguish localized from systemic amyloidosis since patients presenting with localized amyloidosis generally do not need systemic treatment. The most common type of systemic amyloidosis (primary amyloidosis or AL amyloidosis) is caused by production of immunoglobulin light chain or light chain fragment secondary to an underlying plasma cell dyscrasia. Other systemic amyloidoses (secondary amyloidoses) include AA amyloid (caused as a result of chronic inflammatory disease), ATTR caused by alteration in transthyretin (TTR) protein because of one of several mutations (familial amyloid) or misfolding of wild-type transthyretin protein (senile amyloidosis), and dialysis-related amyloidosis (deposition of beta 2 microglobulin).

Primary amyloidosis is the only type of systemic amyloidosis that responds to cytotoxic chemotherapy directed against the abnormal plasma cell clone, the source of amyloidogenic light chains. Other treatment strategies to prevent amyloid formation by altering the equilibrium between soluble precursor and insoluble fibrils, destabilizing the amyloid fibril protein, or antibodies directed against amyloid fibrils are being investigated and show promise but are not available for clinical use [3–5].

This paper will focus on the role of autologous hematopoietic stem cell transplantation for primary systemic amyloidosis.

## 2. Treatment of Amyloidosis

The ultimate goal of treatment of amyloidosis is to improve organ function, prolong survival, and enhance quality of life. The mainstay of treatment is cytotoxic chemotherapy directed against the plasma cells to decrease the level of circulating amyloid causing light chains. This, in turn, can be expected to slow down further amyloid deposition and improve the prospects for mobilization or dissolution of already deposited amyloid and reversal of organ damage. Treatment of AL amyloid has evolved over the years and has closely paralleled the developments in multiple myeloma therapy since the fundamental underlying problem in both these conditions is the same. Early studies established melphalan as an effective agent. In randomized controlled trials comparing melphalan plus prednisone to colchicine in patients with AL amyloidosis, the melphalan and prednisone combination was associated with objective responses and prolonged survival compared to colchicine [6, 7]. Melphalan has continued to be a key agent in the treatment of amyloidosis. Melphalan combined with dexamethasone is considered a “standard” first line therapy. Palladini et al. showed that this combination results in high response rates, 67% hematologic response including 33% complete response, and 48% of patients had objective organ function response. Responses were durable, and CR was maintained in 9 of 15 patients after a median of 4.8 years. Six-year progression-free survival was 40%, and median overall survival was 5.1 years [8, 9]. Other studies with oral or intravenous melphalan combined with dexamethasone have shown less robust responses with complete response rates of 11 and 13%, likely because of different patient characteristics especially cardiac involvement [10, 11]. The development of free immunoglobulin light chain (FLC) assay allowed quantitation of circulating amyloidogenic light chains. Reduction in FLC with treatment was subsequently shown to be associated with decrease in amyloid load, clinical improvement, and survival benefit [12]. The introduction of new agents (IMiDs and bortezomib) in the last 10 years has revolutionized the treatment of multiple myeloma and improved survival [13]. Since the underlying problem in amyloidosis is also plasma cell dyscrasia, it is logical to expect that these agents will also be effective in the treatment of primary amyloidosis. Studies with thalidomide, lenalidomide, and bortezomib indeed demonstrate activity of these agents. Bortezomib appears to be highly effective with hematologic response rates of 50%– 94% and CR rates of 20–44% in different studies [14–16].

## 3. Autologous Stem Cell Transplant (ASCT)

High-dose chemotherapy followed by ASCT reliably produces hematologic complete remissions in a sizable proportion of patients with multiple myeloma. Because plasma cell dyscrasia is the underlying cause of AL amyloidosis, ASCT has also been adopted for the treatment of AL amyloidosis. Early studies of ASCT reported significant toxicity including mortality rate of over 40% in one small study [17]. Subsequent studies showed better results and showed that with careful patient selection transplant can be performed with acceptable toxicity [18, 19]. Autologous stem cell transplant has remained an important treatment option for systemic AL amyloidosis.

### 3.1. Selection of Patients

Although mortality from autologous stem cell transplant has continued to decrease over time, [20, 21] the procedure remains high risk, and careful selection of patients is critical for good outcomes. Best results were obtained in patients with nephrotic syndrome as the predominant manifestation of amyloidosis. Patients with multiple organ involvement, particularly those with cardiac dysfunction, fare poorly with high risk of morbidity and mortality [22]. Many patients with primary amyloidosis are too sick to undergo autologous stem cell transplant.

Predictors of poor prognosis in patients with amyloidosis include the number of organs involved, cardiac involvement evidenced by clinical cardiac dysfunction or elevation of cardiac biomarkers (troponin-T and N-terminal brain natriuretic peptide (NT-pro-BNP)), pretransplant free light chain ratio, and elevated serum uric acid [23–26]. The number of organs involved and the extent of cardiac dysfunction are by far the dominant predictors of prognosis with or without transplant [22, 26]. Excessive fluid retention during stem cell mobilization and harvest may be a reflection of limited cardiac and/or renal reserve and is associated with poor outcomes during transplant [27]. Although older individuals are more likely to have comorbidities, age per se is not a contraindication, and older individuals can successfully undergo autologous HSCT [28]. A risk-adapted approach to transplant has been suggested wherein the melphalan dose in the conditioning regimen is lowered for individuals considered at high risk for transplant toxicity. However, the risk-adjusted lowering of conditioning chemotherapy is associated with reduced response rates [29]. The risks and benefits of transplant have to be carefully evaluated for each individual patient to ensure optimal balance between efficacy and toxicity.

### 3.2. Outcomes

Autologous stem cell transplant is associated with substantial likelihood of hematologic response including organ function improvement. Long-term posttransplant follow-up studies show the durability of responses and better survival of responders. Cibeira et al. reported longterm outcomes of 421 AL patients transplanted between 1994 and 2008. Hematologic CR rate was 43%, and 78% of complete responders had improvement in organ function. For patients achieving a CR the median event free survival (EFS) and overall survival (OS) were 8.3 and 13.2 years compared to organ response rate of 52%, EFS of 2 years, and OS of 5.9 years in those who did not achieve hematologic CR [20]. Investigators from Mayo Clinic reported their experience with 434 transplants since 1996. Hematologic response rate of 76% (CR rate 38.7%) and organ response rate of 46.8% were described. Median OS was not reached for patients achieving hematologic CR and was 107 months and 32 months, respectively, for those with partial response and no response [30]. Outcomes of 107 recipients of ASCT performed at 48 centers between 1995 and 2001 were reported by Center for International Blood & Marrow Transplant Research (CIBMTR) investigators. A relatively high transplant-related mortality of 18% at 30 days was noted. One-year and 3-year survival rates were 66% and 56%, respectively. The outcomes were better in patients transplanted in later years possibly related to better patient selection, improved supportive care and/or physician experience [31]. These and other studies show that hematologic response, particularly CR is associated with better survival and improvement in organ function, including renal and cardiac functions in substantial proportion of patients [32–34]. Histologic regression of amyloid has been shown but seems to occur only in patients who achieve normalization of free light chains [35].

## 4. Hematopoietic Stem Cell Transplant versus Other Therapies

The exact role of autologous stem cell transplant in the treatment of primary AL amyloidosis and the benefit relative to other available therapies continues to be debated. It has been argued that apparent superior outcomes of patients undergoing transplant are a function of selection bias since only healthier patients, who are destined to do better, are selected to undergo transplant. A retrospective study looked at outcomes of patients who were transplant eligible but did not undergo transplant. Transplant eligibility itself identified a good risk group who did better than transplant ineligible patients even with nontransplant therapy [36]. A case control study compared the overall survival of 63 ASCT recipients with matched controls not undergoing transplant. The groups were matched for age, gender, time to presentation, left ventricular function, serum creatinine, inter-ventricular septal thickness, nerve involvement, and 24-hour proteinuria). One-, 2-, and 4-year overall survival rates for the transplant versus non-transplant groups were 89% versus 71%, 81% versus 55%, and 77% versus 41%, respectively [37]. These data showing better overall survival as well as studies demonstrating improved quality of life [38] provide strong arguments in support of the role of autologous HSCT for AL amyloidosis. However, randomized trials have not substantiated the benefit of autologous transplant. In a randomized control trial 100 patients with AL amyloidosis aged 18–70 years were randomized between HSCT and conventional therapy. The overall survival was similar in the two arms [39]. High transplant-related mortality of 24% in this study is a notable concern. The publication generated many letters to the journal highlighting concerns about patient selection, inclusion of high-risk patients which necessitated reduction in melphalan dose in 10 of 37 patients, lack of information about cardiac biomarkers, and whether transplants performed at low volume centers in this multicenter study may have biased the results against ASCT [40–42]. A systematic review and meta-analysis of 1 randomized control trial, 2 other control studies, and 9 single arm studies also did not provide a conclusive answer. The study did not find a survival benefit with transplant but the authors commented that the quality of evidence was low, indicating a need for well-designed and adequately powered trials to better address the role of AHCT in AL amyloidosis [43].

Therapeutic options for AL amyloidosis are constantly changing. As newer, more effective therapies become available, and the role of transplant will need to be reevaluated. Modern, bortezomib-based therapies are associated with response rates approaching those obtained by transplant. Bortezomib as monotherapy or with other agents is active in AL amyloidosis, and produces rapid responses and high rates of hematologic and organ response [44–46]. The durability of these responses is unclear at present since only relatively short followup is available. A phase III, multicenter study evaluating melphalan and dexamethasone with or without bortezomib in patients with previously untreated systemic AL amyloidosis is currently ongoing (NCT01078454). Bortezomib has also been used for amyloidosis relapsing after transplant. It is effective in that setting and can lead to normalization of free light chains and potentially render patients previously not candidates for transplant safe to undergo high-dose therapy [47].

The availability of multiple very active treatment options for patients is clearly welcome news for patients with this devastating disorder. Conventional therapy with bortezomib or other agents and ASCT should not be seen as competitive but rather additive or complementary in nature. Achieving a CR is a key predictor of overall survival. ASCT after a bortezomib-based induction regimen may improve the proportion of patients achieving complete remission. However, that remains to be shown in clinical trials. Amyloidosis is a heterogeneous disease with a large variation in the range and severity of clinical presentations, tempo of disease progression, and comorbidities. The challenge for the physicians is to individualize treatment by selecting the optimal combination or sequence of the various therapies to achieve best possible results for the patient. Clearly, optimal approach will be different for each patient depending on their disease status (number of organs involved, organ function), overall health status and co-morbidities, and their personal choice and goals.

## 5. Conclusions

ASCT is an effective therapy for primary amyloidosis. It is associated with consistently high hematologic response rates, which lead to improvement in organ function, quality of life, and survival. Best results are obtained in patients with 1-2 organ involvement and no cardiac dysfunction. Patients with multiple (more than 2) organ involvement, particularly advanced cardiac involvement and renal insufficiency, are not suitable candidates for this therapy. The question whether ASCT should be the preferred therapy for patients who are healthy enough to tolerate it has not been answered definitely. In the consensus opinion of physicians at the Mayo Clinic experienced in the treatment of amyloidosis the answer to this question is yes [48]. The importance of patient selection to ensure safety and optimal outcomes cannot be overemphasized. The role of HSCT in the overall treatment of AL amyloidosis is likely to evolve as new, more effective therapies become available and will need to be continually assessed in future prospective trials.
